# Experiences with insecticide-treated curtains: a qualitative study in Iquitos, Peru

**DOI:** 10.1186/s12889-016-3191-x

**Published:** 2016-07-16

**Authors:** Valerie A. Paz-Soldan, Karin M. Bauer, Audrey Lenhart, Jhonny J. Cordova Lopez, John P. Elder, Thomas W. Scott, Philip J. McCall, Tadeusz J. Kochel, Amy C. Morrison

**Affiliations:** Department of Global Community Health and Behavioral Sciences, Tulane University School of Public Health and Tropical Medicine, 1440 Canal Street, Suite 2200, New Orleans, LA USA; Facultad de Salud Pública y Administración, Universidad Peruana Cayetano Heredia, Lima, Peru; Entomology Branch, Division of Parasitic Diseases and Malaria, United States Centers for Disease Control and Prevention, Atlanta, GA USA; Department of Vector Biology, Liverpool School of Tropical Medicine, Liverpool, UK; Division of Health Promotion and Behavioral Sciences, Graduate School of Public Health, San Diego State University, San Diego, CA USA; Department of Entomology and Nematology, University of California Davis, Davis, CA USA; Fogarty International Center, National Institutes of Health, Bethesda, MD USA; Virology Department, Naval Medical Research Center, Silver Spring, MD USA

**Keywords:** Dengue, Insecticide treated curtains (ITC), Peru, Qualitative

## Abstract

**Background:**

Dengue is an arthropod-borne viral disease responsible for approximately 400 million infections annually; the only available method of prevention is vector control. It has been previously demonstrated that insecticide treated curtains (ITCs) can lower dengue vector infestations in and around houses. As part of a larger trial examining whether ITCs could reduce dengue transmission in Iquitos, Peru, the objective of this study was to characterize the participants’ experience with the ITCs using qualitative methods.

**Methods:**

Knowledge, attitudes, and practices (KAP) surveys (at baseline, and 9 and 27 months post-ITC distribution, with *n* = 593, 595 and 511, respectively), focus group discussions (at 6 and 12 months post-ITC distribution, with *n* = 18 and 33, respectively), and 11 one-on-one interviews (at 12 months post-distribution) were conducted with 605 participants who received ITCs as part of a cluster-randomized trial.

**Results:**

Focus groups at 6 months post-ITC distribution revealed that individuals had observed their ITCs to function for approximately 3 months, after which they reported the ITCs were no longer working. Follow up revealed that the ITCs required re-treatment with insecticide at approximately 1 year post-distribution. Over half (55.3 %, *n* = 329) of participants at 9 months post-ITC distribution and over a third (34.8 %, *n* = 177) at 27 months post-ITC distribution reported perceiving a decrease in the number of mosquitoes in their home. The percentage of participants who would recommend ITCs to their family or friends in the future remained high throughout the study (94.3 %, *n* = 561 at 9 months and 94.6 %, *n* = 488 at 27 months post-distribution). When asked why, participants reported that ITCs were effective at reducing mosquitoes (81.6 and 37.8 %, at 9 and 27 months respectively), that they prevent dengue (5.7 and 51.2 %, at 9 and 27 months), that they are “*beautiful*” (5.9 and 3.1 %), as well as other reasons (6.9 and 2.5 %).

**Conclusion:**

ITCs have substantial potential for long term dengue vector control because they are liked by users, both for their perceived effectiveness and for aesthetic reasons, and because they require little proactive behavioral effort on the part of the users. Our results highlight the importance of gathering process (as opposed to outcome) data during vector control studies, without which researchers would not have become aware that the ITCs had lost effectiveness early in the trial.

## Background

With an estimated 96 million apparent (or symptomatic) infections and an additional 294 million inapparent (or “asymptomatic”) infections globally in 2010, dengue viruses (DENV) are the cause of more human morbidity and mortality than any other arthropod-borne virus [1]. The public health burden of dengue in the Americas is particularly high with 2.3 million dengue cases reported in 2013, the greatest number to date [2]. Peru is one of the countries in the Americas that is at greatest risk, reporting nearly a third of the region’s dengue cases in 2013 [3]. *Aedes aegypti* and *Aedes albopictus* mosquitoes, dengue virus vectors in Latin America, also transmit Chikungunya and Zika viruses, which are increasing as well [4, 5].

Currently, vector control is the only tool available to prevent and control dengue transmission (as well as Chikungunya and Zika), as vaccines and anti-viral medications remain in development and testing phases [6–8]. Most vector control strategies target immature *Aedes aegypti* mosquitoes, typically in containers used for water storage near the human habitations where they proliferate; such strategies only decrease mosquito vector density and do not reduce the longevity of adult mosquitoes [9]. While interventions using insecticide sprays that target adult *Ae. aegypti* do exist, they are labor intensive to deliver inside homes where the mosquitoes live and typically only kill mosquitoes during a short time period (i.e., they have limited efficacy and residuality), and are therefore used primarily in response to outbreak situations [10–13]. Insecticide-treated curtains (ITCs) and insecticide-treated screens can be an effective household-level *Ae. aegypti* control measures that reduce the number of adult mosquitoes in homes [14–18]. A challenge with the approach, however, is that their use can significantly decline in the long-term [19, 20]. A study in Venezuela and Thailand found that household-use decreased to 59.7 % after 18 months, and further declined to 38.4 % after 22 months [21]. In order for ITCs to be an effective vector control tool, it is imperative to understand why people do or do not accept and continue to utilize ITCs over time.

Many quantitative and qualitative studies have been conducted about the use of insecticide-treated materials (ITMs) as vector-control interventions for malaria prevention, such as bed nets and durable wall linings [22–29], yet little research has been conducted on the acceptability of ITCs as a vector control intervention for dengue and other viruses transmitted by *Aedes*. One dengue prevention study conducted in Venezuela and Thailand found that participants were more likely to accept and use ITCs if the curtains were perceived as being effective [19], although a qualitative component to understand why people did or did not like ITCs was not included in that study. Patients’ compliance (or lack of) is an underestimated aspect of vector control measures, and can result in poor evaluations of the effectiveness of a given intervention.

As part of a larger cluster randomized trial examining whether ITCs could reduce *Ae. aegypti* populations and DENV transmission in Iquitos, Peru, the objective of this study was to qualitatively characterize participants’ experience with the ITCs during the trial. Related to this study, other analyses have been conducted focusing on this population’s overall knowledge, attitudes, and dengue prevention practices, as well as factors associated with correct and consistent use of the ITCs (based on the research team’s curtain monitoring), which were recently published [20, 30]. This manuscript specifically focuses on the experiences with the ITCs by those who had the ITCs in their homes: i.e., what they liked, what problems they had. By understanding what participants like or do not like about ITCs, ITC intervention programs can develop more effective strategies for community acceptance and sustained proper use of this tool for dengue prevention.

## Methods

### Study setting

Iquitos, Peru is a large (population ~400,000), geographically isolated city in the Amazon Basin that is only accessible by boat or plane [31]. This study was conducted in the southern-most extent of the urban part of the city located in the district of San Juan. There is excellent infrastructure for monitoring local dengue transmission and vector populations because extensive dengue epidemiology studies have been conducted in Iquitos since 1999 by the University of California at Davis/U.S. Naval Medical Research Unit-6 (NAMRU-6) [20, 30, 32–41]. As such, experienced research teams are in place to collect and monitor entomological and serological data, as well as collect and analyze quantitative and qualitative data related to people’s knowledge, beliefs and practices associated to preventing dengue and other vector-borne diseases.

### Study design

This study was part of a cluster-randomized controlled trial, initiated in October 2009, to measure whether ITCs could reduce DENV transmission and mosquito vector density in 10 treatment clusters compared to ten control clusters of approximately 70 households each (2–3 city blocks). The ITC intervention consisted of distribution and installation of PermaNet 2.0 (Vestergaard, Lausanne, Switzerland) curtains [42], factory treated with a long lasting formulation of the insecticide deltamethrin. Serology samples and entomological surveys were collected every 9 and 6 months, respectively, both in the intervention and control clusters. Weeks prior to ITC distribution, we asked one resident from each intervention and control household to participate in a survey about knowledge, attitudes and practices (KAP) associated with dengue and mosquito control [30]. The ITCs were then distributed to 593 intervention households between November and December 2009. Follow up KAP surveys, interviewing the same individuals that had been interviewed previously (with up to eight visits at mornings, afternoons or evening hours to find that same individual), were carried out at 9 and 27 months post-ITC distribution, and a curtain monitoring checklist was conducted at 9 and 18 months post-distribution [20]. After the initial distribution of ITCs, 12 households in the intervention cluster became interested in participating after seeing the ITCs; hence, at 9 months, there were 605 intervention households, and at 27 months there were 516 intervention households. Data used for this analysis comes from the 593 intervention households that participated in the baseline KAP, and the 595 and 511 intervention households, from months 9 and 27 respectively, with complete data for all the variables in this analysis.

Focus group discussions (FGDs) were conducted at 6 and 12 months post-distribution, and one-on-one interviews at 12 months post-distribution. During the 2 FGDs at month six post-distribution, participants (*n* = 18) reported the ITCs had stopped killing insects after ~3 months. A sample of ITCs were pulled from households and the efficacy of the deltamethrin was tested using standard WHO cone bioassays [43]. Results from the cone bioassays showed reduced bioefficacy of the ITC material, consistent with reports during FGDs that the ITCs seemed to stop working after ~3 months. Hence, at 12 months into the study, ITCs were removed from homes, re-treated with deltamethrin (K-O tab, Bayer), and returned to their original location in the homes.

### Surveys

A baseline KAP survey of the households was conducted between October-December 2009 – prior to and during the time of ITC distribution. We asked to interview the person who would be managing the ITCs and makes related decisions in the home, which was usually the housewife. The first KAP survey that included questions regarding the participants’ experience with the ITCs was conducted after the ITCs had been in place for 9 months, between July–October 2010—this survey was conducted with the same individual interviewed at baseline (after 8 unsuccessful attempts to find the person, we interviewed another household decision maker who also took care of the home and the ITCs). The final KAP survey including a section on the participants’ experience with the ITCs was conducted 27 months after initial distribution (February–April 2012), again, with the same individual who had responded to previous KAP surveys (and again, after eight unsuccessful attempts, we interviewed another household decision maker who cared for the home and the ITCs). A few observations were missing responses to some of our key variables of interest for this manuscript, so we analyzed data from the individuals from intervention area for whom we had complete KAP data at 9 months (*n* = 595), and at 27 months (*n* = 511). Written consent was obtained from all who participated in the KAP surveys at baseline, 9 and 27 months post-distribution.

The KAP surveys were developed, piloted, and modified by the lead social scientist working with the research team in Iquitos. This also served as training for the research assistants and ensuring consistency in documenting responses between them. Most of the questions were closed-ended, except for a few, including: for how many months did the ITCs seem to work, why would you recommend the ITCs to someone else in the future, and how many ITCs would you request in the future if you could pick a number again. Responses to these questions were coded and categorized based on themes that came up, whereas responses to the closed-ended questions had been developed based on preliminary work piloting the surveys.

### Focus group discussions and interviews (Table 1)

**Table 1:** Summary of focus group discussions and interviews conducted in Iquitos, Peru

Group	No. FGD/Interviews	No. Participants
Focus groups – 6 months after distribution	2	18
Focus groups – 12 months after distribution	3	33
One-on-one interviews – 12 months after distribution	11	11
Total	--	62

Convenience sampling was used to select FGD participants. Specifically, due to our experience with low turn out when setting up times in advance for FGD, on the day of the FGD, about 2 hours prior to the scheduled time, our field teams went to 3 intervention blocks that were near to one another (to make picking participants up for transport to the FGD site easier), and described to people they found in their homes about the FGD and if they would be willing to participate. Three assistants—one at each block—each were asked to recruit 3–4 people, with the instruction of ensuring that the houses had at least 4 houses of distance from one another. Assistants stopped mototaxis/rickshaws to take the FGD participants to the FGD site, where 2 FGDs with 18 individuals were conducted in January 2010, 6 months after initial ITC distribution. Three additional FGDs (*n* = 33 individuals) were conducted in June 2011 (12 months after initial ITC distribution and following ITC re-treatment with insecticide). In June 2011, 11 one-on-one interviews were also conducted with participants who had rejected the ITCs after having them in their home for at least a week.

The FGD facilitator and interviewer of the one-on-one interviews was a Peruvian social scientist experienced at facilitating FGDs and interviews on vector-related topics in Iquitos. The emphasis of the FGDs conducted 6 months post-ITC distribution was to ascertain where the ITCs had been hung in the home, ask about the advantages and disadvantages of each location, obtain overall comments from participants about their experiences with the ITCs up to that time, and allow the participants to ask questions related to the ITCs. Participants were asked if they experienced any issues with the ITCs and if they would accept ITCs again. During the FGDs held at 12 months, participants were asked about changes in utility of the ITCs since their initial distribution or after the first re-treatment with insecticide. The FGD at 12 months also included a discussion on the participants’ reaction to the need for re-treatment. The eleven one-on-one interviews were semi-structured and focused on exploring the reasons why the participants had rejected having the ITCs in the home.

Informed verbal consent was obtained from all participants. Detailed notes were taken during the FGDs and interviews by study personnel. After the FGDs and the interviews, the note-taker who was recording during the FGDs and other research team members compared their notes to ensure inclusion of all notes and comments in the final written documentation of the FGDs. Digital recordings were made of the FGDs to aid with the writing of the detailed documentation, but were not transcribed.

### Data analysis

Responses from the KAP surveys were analyzed using STATA 12.0 [44]. Frequencies for close-ended questions were tabulated; responses to open-ended questions were coded manually and then tabulated.

Open ended responses to survey questions were synthesized into categories for quantification purposes. For example, when asked in an open-ended question why they would or would not recommend ITCs to their family or friends at 9 months after ITC distribution, answers were collapsed into the four main themes that emerged: prevents dengue, reduces the amount of mosquitoes, ITCs are beautiful, and other. The category “prevents dengue” included those who responded that the ITCs specifically prevented dengue or protect one’s health. The category “reduces the amount of mosquitoes” included the following participant responses: protects against mosquitoes, reduces the amount of mosquitoes, kills mosquitoes, kills all insects, or feels the ITCs are effective. The category “ITCs are beautiful” included those who responded to the aesthetic properties of the ITCs, i.e., “beautiful”, “decorative”, “to adorn one’s house” or “elegant”. The category “other” included all other participant responses, including “privacy”, “security”, “free”, or “economical”. FGD and interview data was coded manually and summarized based on themes of interest.

### Ethics statement

This trial received approval from the Institutional Review Boards (IRBs) at the Liverpool School of Tropical Medicine (09.59), the Tulane School of Public Health and Tropical Medicine (166680–4), and the U.S. Naval Medical Research Unit-6 in Peru (NMRCD.2009.0007). The Tulane School of Public Health and Tropical Medicine and the University of California at Davis also had an inter-institutional IRB agreement with the U.S. Naval Medical Research Unit-6. The Loreto Regional Government was also informed of this study in writing, and gave their approval for this study to proceed (letter #586-2009-GRL-DRS/30.09.01). Written consent was obtained for participants who participated in the surveys, and verbal consent was obtained from each participant in the FGD for: 1) voluntary participation in the FGD and 2) having the FGD audiotaped.

The trial was registered with the International Standard Randomized Controlled Trial Number Register: ISRCTN08474420. This manuscript does not report the outcome of the cluster randomized trial. The investigation was carried out only on the treatment group; the control group in the study received no treatment. Therefore this study is not an analysis that is dependent on randomized control trial (RCT) design, but instead measures perceptions of the intervention tool: the ITCs.

## Results

### KAP participant characteristics

The median age of KAP participants in the intervention clusters was 39 years and most participants were female (76.7 %). Almost 80 % of participants had less than or equal to 11 years of education and the primary occupation was housewife (48.7 %). The median number of people living in the households was five, including adults and children. One fifth of houses had a child under the age of three living in the home. Each household decided on the number of ITCs they would receive: the median number of ITCs distributed per house in this study was five, with a range of 1 to 15.

### KAP surveys

#### Perceived effectiveness

At 9 months post-ITC distribution, over half of participants responded that they saw a drop in the amount of mosquitoes in the home (the proxy variable for perceived effectiveness), but a third of participants responded that they saw a drop only for a few months (Table 2). By 27 months after distribution, the percentages had switched so that over half of the participants reported seeing the drop for only a few months and only a third reported seeing a decrease in the amount of mosquitoes throughout the study period. The average number of months that the ITCs were reported as functioning was 3.3 months and 4.7 months at 9 months and 27 months, respectively (Table 2).

**Table 2:** Participant perception of ITC effectiveness and future use at 9 and 27 months after initial distribution in Iquitos, Peru (KAP surveys: *n* = 595 at 9 months, *n* = 516 at 27 months)

	9 months % (*n*)	27 months % (*n*)
Saw a decrease in the amount of mosquitoes		
No	9.8 (58)	9.6 (49)
Yes, but only for a few months	35.0 (208)	55.7 (285)
Yes	55.3 (329)	34.8 (177)
Doesn’t know		1.0 (5)
Average number of months that the ITCs functioned (at 9 mos: *n* = 205; at 27 months: *n* = 284)^a^	3.3 months (range 1--8)	4.7 months (range 1--24)
Percent who would recommend ITCs to family or friends	94.3 (561)	94.6 (488)
If they saw a decrease in mosquitoes (even if just for a few months), would they recommend ITCs?	97.8 (525)	97.0 (462)
If they did not see a decrease in mosquitoes, would they recommend ITCs?	62.1 (36)	75.7 (37)
Reasons for recommending ITCs^b^ (at 9 months: *n* = 561; at 27 months: *n* = 488)		
Reduces the amount of mosquitoes	81.6 (460)	37.8 (195)
Protects against dengue	5.7 (32)	51.2 (264)
Beautiful/aesthetically pleasing	5.9 (33)	3.1 (16)
Other	6.9 (36)	2.5 (13)
Reasons for not recommending ITCs^b^ (at 9 months, *n* = 34; at 27 months, *n* = 28)		
Did not see a decrease in mosquitoes	32.3 (10)	39.3 (11)
ITCs “not effective”	45.2 (14)	35.7 (10)
Other	22.6 (7)	17.9 (5)
No response	9.7 (3)	7.1 (2)
What they liked about the ITCs		
Saw fewer mosquitoes in the home	44.0 (262)	36.8 (190)
Looked beautiful/elegant	25.4 (151)	27.3 (141)
Saw dead mosquitoes on the ITCs	15.8 (94)	24.6 (127)
Saw all types of dead insects dead on the ITCs	9.9 (59)	7.4 (38)
Other	4.4 (26)	3.7 (19)
Missing	0.5 (3)	0.2 (1)
What problems were experienced with the ITCs		
No problems	78.0 (464)	77.0 (397)
Allergic skin reaction	19.2 (114)	20.2 (104)
Respiratory reaction	3.2 (19)	2.1 (11)
Got easily dirty/damaged where they were located	0.5 (3)	0.2 (1)
Interfered with movement in the house	0.2 (1)	0.6 (3)

^a^There were three outliers in the 9 months survey (who responded “15 months” or “24 months” when ITCs had only been out for 9 months), and one respondent who “did not know” how many months the ITCs had worked well in the 27 months survey. These four outliers were removed from the calculation of the mean

^b^Participants were asked to respond to these open-ended questions in the 9 month KAP survey and the responses were collapsed into these four categories

#### Reasons for recommending ITCs

The majority of participants at 9 and 27 months after distribution responded that they would recommend the ITCs to family or friends: 94.3 % at 9 months and 94.6 % at 27 months (Table 2). Approximately 97 % of those who perceived a change in the number of mosquitoes in their home, even if for just some months, would recommend the curtains (both at 9 and 27 months). Interestingly, 62.1 and 75.7 %, at 9 and 27 months respectively, would recommend the use of ITCs even if they did not perceive a difference in the number of mosquitoes in their homes.

Participants were asked to elaborate through an open ended question why they would or would not recommend ITCs and answers were recorded, coded, and based on the themes that emerged, collapsed into four categories. At 9 months, the majority (81.6 %, *n* = 460) of those who would recommend ITCs reported it was because they reduce the number of mosquitoes; 5.7 % (*n* = 32) of participants specifically responded that the ITCs prevented dengue, which was the primary aim of the ITCs in this study. However, at 27 months, more than half (51.2 %, *n* = 264) of participants reported they would recommend ITCs because they protect against dengue and other illnesses. It is important to note that many participants’ first response was that the ITCs reduced the number of mosquitoes (the proxy variable for perceived effectiveness), but these responses were quickly followed by stating that ITCs were not long-lasting in their protection. Other reasons given for why ITCs would be recommended included that they provided security, privacy (i.e., some ITCs were used as doors to bathrooms or bedrooms since some houses did not have doors) and “overall protection” for the family (not specifying for what or how). Additionally, participants mentioned that the ITCs were good because they were more economical than other mosquito control measures, as they were given free of charge to study participants.

Among those who would not recommend ITCs (*n* = 34 at 9 months and *n* = 28 at 27 months), the top two reasons cited for why they would not recommend ITCs were that the ITCs were not effective (45.2 and 35.7 %, at 9 and 27 months, respectively) and that they perceived no difference in the amount of mosquitoes in the home (32.2 and 39.3 %, at 9 and 27 months, respectively) (Table 2).

#### Likes and dislikes

The participants were asked what they liked most about the ITCs (Table 2). The top three items reported at 9 and 27 months was seeing fewer mosquitoes in their homes (44 and 36.8 %, at 9 and 27 months, respectively), feeling that the ITCs were beautiful/elegant (25.4 and 27.3 %, respectively), and seeing mosquitoes and other insects dead on the ITCs or the floor underneath. When asked if they had any problems with the ITCs, over three quarters of participants reported “none” at 9 and 27 months. The most common problem reported was an allergic skin reaction (19.2 %, *n* = 114 at 9 months, 20.2 %, *n* = 104 at 27 months), with a small percentage reporting a respiratory reaction (3.2 % *n* = 19 at 9 months, 2.1 %, *n* = 11 at 27 months).

#### Future curtain use

**Fig. 1:**
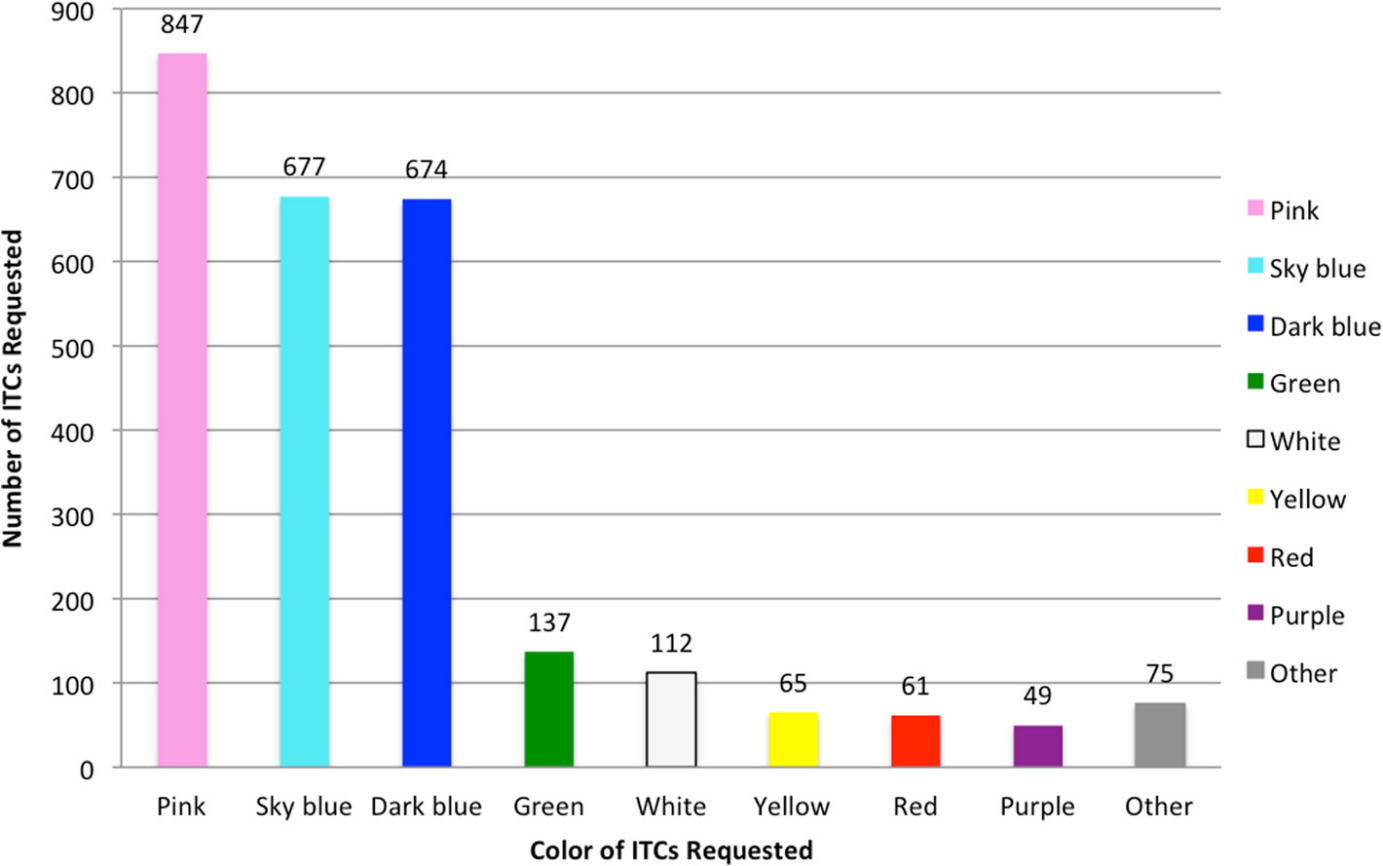
Number of ITCs requested by color, 27 months after distribution in Iquitos, Peru. (*n* = 2697)

**Fig. 2:**
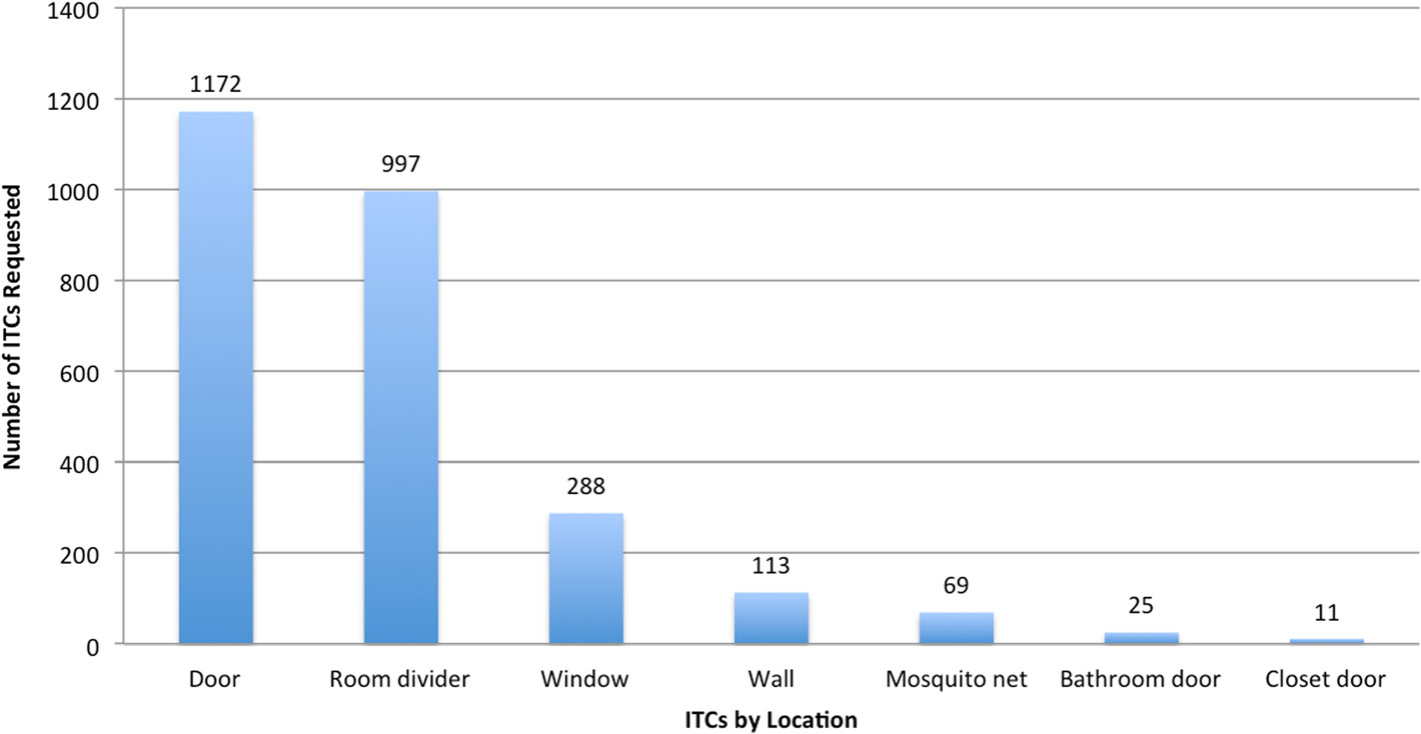
Number of ITCs requested by location, 27 months after distribution in Iquitos, Peru. (*n* = 2675)

At 27 months, the majority of participants (85.3 %, *n* = 440) responded that they would select ITCs again at the end of the study. A median of five ITCs would be requested per household (range 1–29), which is equal to the median number of ITCs that were requested and distributed per household in this study. Pink was the most favored color (31.4 %, *n* = 847), followed by sky blue (25.1 %, *n* = 677) and dark blue (25.0 %, *n* = 674) (Fig. 1). Most participants reported wanting to place their ITCs in the door (43.8 %, *n* = 1172) or as a room divider (37.1 %, *n* = 997) (Fig. 2), despite the fact that we found that ITCs placed in these locations were most likely to be tied up during the day, whereas those on walls were most likely to remain in place and out of the way throughout the day [20]. It is important to note that the questions regarding preferred ITC color and placement location for ITCs were open ended, as was the question regarding how many ITCs they would select, and when respondents answered to the three separate questions, the numbers for the ITCs did not add up, hence the difference in ITCs in Fig. 1 (*n* = 2697) and Fig. 2 (*n* = 2675).

#### Other mosquito control products

At baseline, when asked about products used for mosquito control, most reported using mosquito nets (84 %); petroleum, creoline or bleach on their floors (45.2 %), and insect sprays or slow-burning insecticide coils (36.7 %). Moreover, related to external interventions (usually carried out by the local Ministry of Health), fumigation and larviciding was reported in 53.1 and 58 % of the houses, respectively. Most participants at 9 and 27 months post-ITC distribution reported using the same amount of certain mosquito control products over the preceding 9 months. However, there was a reported reduction in use of products at both time periods: when asked about specific products, and if they were using them more, less, or in the same amount compared to 9 months prior, 17.5 % reported using their mosquito nets less; 38 % reported using less of the petroleum, creoline, or bleach on their floors; and 55.6 % reported less use of insecticide sprays or the slow-burning insecticide coils. Reported use of these products decreased further at 27 months: 12.2 % reported using mosquito nets less; 15.8 % reported using less of the petroleum, creoline, or bleach on their floors; and 36.7 % reported less use of insecticide sprays or the slow-burning insecticide coils. At the 9 months survey, participants also reported that there had been less fumigation/larviciding in their homes in the previous 9 months (~20 %), and at the 27 months survey, ~10 % reported less fumigation/larviciding in their homes in the previous 9 months. The main reason given for reducing their use of all products was that they perceived a drop in the amount of mosquitos in the home.

### Focus groups discussions and interviews

#### Advantages and disadvantages at 6 months

The two FGDs at 6 months post-distribution were conducted only with women, because they reported that they were the ones in charge of managing their homes and making decisions regarding whether to and where to hang ITCs. Participants liked the ITCs because they saw them working instantly: *“They work well: you could see all kinds of dead insects at the bottom of the ITCs, even cockroaches and spiders….”* The enthusiasm among all participants was clear. However, very early on in the discussion of both FGDs, one participant mentioned, *“They worked well for a few months, but then they stopped working. You could see dead insects on the floor of your house when we first placed the ITCs, but then after a few months, you didn’t see any dead insects any more…”* Once one woman raised the issue, all the other participants agreed; this happened independently in both FGDs, resulting in the women reporting that the ITCs were effective, but only for 2–6 months after the initial distribution. As a result of the ITCs’ initial effectiveness, the participants commented that they decreased use of other mosquito repellents, no longer had to use bed nets at night because there were fewer mosquitoes in the home, or stopped fumigating: *“After 2 months they came to fumigate and I didn’t allow them to because it was no longer necessary…because I had the curtains.”*

The participants perceived the ITCs to be *“beautiful”* and noted that they would continue to hang the ITCs in their homes for decorative value, even if they were no longer effective at controlling mosquitoes. Some participants reported that family and friends were envious of the ITCs and asked if they could borrow the ITCs. One participant told of loaning her ITCs to a relative organizing a birthday party for her child in order to beautify her home. The ITCs were also reported to provide additional privacy in the home, particularly as a “*separator between beds, it gives privacy.”* At this point in time, most ITCs did not require mending (only six had been mended, albeit 5.5 % (159) of the ITCs were observed by our research team to be in poor state [20]) and participants reported that all colors of the ITCs were working equally well at controlling mosquitoes. There were also placement preferences based on ITC colors and how these were perceived: the pink ITCs were considered more “*elegant*” by FGD participants and were selected for placement in locations such as their living rooms, whereas the dark blue ITCs were harder to see through and, hence, selected for locations where the participants might want more privacy, such as bathroom or bedroom doors.

Similar to the findings from surveys, there were few ‘dislikes’ reported. The main problems included that the ITCs were initially itchy or stingy on the skin because of the insecticide (but that itchy feeling had disappeared after the first week), that the ITCs could not be moved once placed because the study team told them not to move the ITCs, and that the ITCs got dirty quickly and could not be washed quickly enough to keep clean: “*Both colors (pink and blue) get equally dirty -- they get dirty quickly.*”

All participants stated they used the ITCs in the way they were expected to (i.e., left hanging loosely), but in every group several women mentioned tying up the ITCs during certain times of the day to avoid physical contact with the ITCs, particularly if the ITCs were hung in the door or passageway. One mentioned, for example,*“I have to tie it up during the day because it bothers the clients who come to my house for my photocopy business”* (the ITC is near the machine). She would then let it hang loosely again in the evening and overnight. Some felt that the wear and tear on some ITCs was high due to all the movement in certain locations of their home.

The FGD participants also noted creative uses for the ITCs, which were not consistent with the study instructions. For example, multiple participants reported turning the ITC into a bed net to sleep under (either for themselves or children): *“it is safer as a mosquito net – it is better because it protects more at night”.* One participant took her ITCs back and forth with her to her farm on the outskirts of Iquitos because she wanted the protection of the ITCs while at her farm.

Information arising from this set of FGDs regarding a perceived drop in the effectiveness of the ITCs led to an increase in the number of curtains that were tested for insecticide bioefficacy between 6–12 months post-distribution. These FGD data were the first indication that the ITCs were not functioning as expected, and indeed bioefficacy testing corroborated the FGD data. As a result, all ITCs were collected after 12 months of distribution and were re-treated with insecticide, and then returned to the houses.

#### Advantages and disadvantages at 12 months

A further three FGDs were conducted with female participants 12 months post-ITC distribution. These FGDs aimed to understand how the ITCs were functioning immediately after the re-treatment with insecticide. All of the FGDs reported the ITCs worked best in the first few months of use, when they came from the manufacturer; however, all re-treated ITCs worked better than they had prior to re-treatment.

The comments about how the ITCs worked after re-treatment were different than the comments in the first set of focus groups. At 12 months, the participants noted that *“the amount of mosquitoes only went down the first time,”* but they did not notice a drop in mosquitoes after the second re-treatment. Mosquitoes were reported as being stuck to the ITCs after re-treatment, though they were still alive on the ITCs rather than being dead like when the ITCs were new. The participants also commented that the ITCs had a strong smell and burned the skin after re-treatment. There were also complaints about the re-treatment process, because it required study personnel to come repeatedly to the house; it would have been preferable if study personnel had arranged set appointments to pick up their curtains for re-treatment.

When asked if they would rather have future ITC re-treatments performed inside or outside the home (i.e., rather than removing ITCs and re-treating them off-site, have a research team member re-treat the ITCs at the participants’ homes), there were mixed responses. Some participants preferred that it occurred outside the home due to the believed toxicity of the insecticide and the desire to protect their families’ health. Others wanted the treatments to be done inside “s*o I can see how it’s done and do it myself one day.”*

Other comments during the FGDs highlighted the fact that the ITCs had come to be valued by the participants as a means to protect against dengue. They reported that fear of dengue was a motivator for hanging the ITCs correctly, “*I’m more attentive to using [the ITC] after the second re-treatment, in case of a dengue outbreak.”* Though others noted that they still had to pull the ITCs aside during the day due to fear of allergies and to allow transit in their homes.

#### One-on-one interviews

One-on-one interviews were only conducted with participants who had rejected or returned their ITCs. The reasons for returning their ITCs were similar to those disadvantages listed during the FGDs. The main concerns were allergic reactions, inconvenience of the study staff coming to re-treat the ITCs, and the ITCs’ loss of effectiveness.

Four of 11 interviewees reported allergic skin reactions as the reason for rejection. For example, one interviewee reported that she loved having the ITCs in her home because they were pretty and there were less mosquitoes, but that her two young grandchildren had many allergies and their skin became irritated when they walked by the ITCs. She would have liked to keep the ITCs, but felt that she had no option but to return them because of the allergic reaction of her grandchildren.

Another interviewee mentioned that she had turned the ITC into a bed net for her baby and did not want the study staff re-treating it because she feared that the insecticide would give the child an allergic reaction. Three interviewees mentioned being inconvenienced by study personnel constantly coming to the door for follow-up visits and re-treatment of the ITCs, as well as the short term effectiveness of the ITCs.

Five interviewees reported that the ITCs were no longer effective. One interviewee reported that the ITCs were not effective even after the re-treatment and that there were many more mosquitoes in her home now. Another interviewee reported that the ITCs had to be tied up all day due to their location in the hallway and she concluded that they were not working well enough for her to keep them.

## Discussion

In the surveys, focus group discussions and one-on-one interviews, participants reported similar likes and dislikes related to the ITCs. The participants perceived that the ITCs were effective (though the effectiveness was not long-lasting), decreased the amount of mosquitoes, and were aesthetically pleasing. These reported advantages are consistent with studies of insecticide treated nets (ITNs) for malaria prevention conducted in Western Kenya and rural Malawi. The Kenya study compared curtains to bed nets for malaria control and, while overall bed nets were preferred over the curtains, the study participants did report that the advantages of the curtains included that they prevented mosquitoes from entering the home and that they enhanced the beauty of the home [28]. However, while the Malawi study found that participants associated the curtains with reductions in malaria transmission [29], our study did not consistently show that participants connected the ITCs with dengue prevention.

The amount of participants who reported they would recommend ITCs in the future because they prevented dengue increased from 5.7 % at 9 months to 51.2 % at 27 months after ITC distribution. The increase could be because the participants learned about the purpose of the ITCs during repeated visits to their homes for re-treatment by study staff, despite the fact that they were told at the start of the study that we were testing whether the ITCs could prevent dengue. Alternatively, this may reflect the shift from dengue serotype 4 (August 2008-early 2011) to dengue serotype 2 transmission in Iquitos [45–47]. The latter serotype was first observed in late 2010, causing a dramatic epidemic with a much higher proportion of severe disease than observed during dengue-4 transmission [45–47]. This dengue outbreak was highly publicized and overwhelmed local hospitals, and it occurred between the 9 and 27 months KAP surveys. Related to these issues, when asked this question, the respondents’ first answer was documented, so if respondents had been able to provide more than one answer, they might have mentioned dengue prevention as a reason to recommend ITCs at both time points. The shift may reflect a shift in prioritization rather than a shift in awareness.

There were remarkably few ITC dislikes reported during FGDs. More than three quarters of participants reported that they did not have any problems with the ITCs. The most commonly reported dislike was allergic skin reactions, which were typically described to occur in the first week or two following placement or re-treatment. This finding was consistent with the one-on-one interviews in which a third of interviewees rejected the ITCs due to reactions to the insecticide. The FGDs highlighted that they did not like that the ITCs got dirty so quickly, had to be washed regularly, and that they could not be moved once placed by the study personnel. They similarly disliked that the ITCs had to be re-treated and even then were not perceived to be as effective as when they were brand new. These findings are similar to a study of insecticide-treated bed nets conducted in Ethiopia where participants reported that they did not use the ITNs because they had lost effectiveness [25].

The perception of effectiveness has been shown as important for acceptability of nets and curtains in other studies of vector borne diseases. In the Solomon Islands, the main criterion for acceptability of bed nets was effectiveness at preventing mosquito bites [22]. A study conducted in five malaria-endemic areas in three countries of South America also found the acceptability of nets was related to participants’ perception of protection against mosquitoes [48]. In a different analysis of the Iquitos data, we also found that the odds of recommending ITCs in the future were significantly greater among those who perceived the ITCs to be effective [20]. Results from this study demonstrated that participants accepted the ITCs even though their perception of effectiveness of ITCs was short-term. The total percentage of participants who would recommend ITCs to family or friends in the future remained strong at 9 and 27 months after distribution (94.3 and 94.6 %, respectively), even though the percentage of participants who perceived that the ITCs only functioned for a few months increased from 35 to 55.7 % at months 9 and 27, respectively. Basically, if an effective and long-lasting ITC were available, acceptability for these would be very high and could be a good alternative or supplement to other vector control activities.

The participants reported in the surveys and the FGDs that they perceived the ITCs to be attractive and that they would even keep the ITCs for decorative purposes even if they ceased to be effective at controlling mosquitoes. These findings are consistent with others in the literature. A study of a durable wall lining for malaria prevention in Africa and Southeast Asia found that 66 % of participants would keep their wall lining for decorative purposes even if it lost its effectiveness [24]. Participants also reported that they would select to have ITCs again (median = 5), with most preferring pink or sky blue ITCs because these were considered the most “elegant” and one quarter preferring dark blue ITCs because these offered most privacy (i.e., these were often placed in doorways). A malaria prevention study in the Solomon Islands also found that dark colored insecticide-treated nets were preferred by participants for privacy [22]. Therefore, the aesthetic qualities of any vector control material should always be a consideration.

Creative or incorrect use of nets has been documented in other studies. In a study of bed nets for malaria prevention in the Solomon Islands, participants were reported as using the nets for fishing or protecting crops [22]. Similarly, a study of ITNs for malaria prevention along the shores of Lake Victoria in Kenya found that participants were using the nets for drying fish and for fishing [49]. Participants in the Iquitos FGDs also reported that the ITCs were used incorrectly or creatively at times during our study, such as turning them into bed nets for children or for other family members to sleep underneath. This demonstrates that either protection from nuisance night-time biting mosquitoes was the priority for some, or that the participants did not understand that the dengue mosquitoes only bite during the daytime and, therefore, the ITCs should be hung up in the home during the day, not used as nighttime to protect against night-time biting mosquitoes [30]. This conclusion is consistent with results reported in another manuscript revealing that only 18.6 % of participants knew that *Ae. aegypti* bites during the day or early evening [30]. When the team’s social scientist, who is from Lima, mentioned this to several people during follow up interviews, she was told by several that, *“In Lima the mosquitoes might bite during the day time, but in Iquitos, they bite at night.”*

### Limitations

The participant population was primarily female, consequently we have little feedback from men regarding the ITCs. However, in Iquitos, women are mainly responsible for household management and they were the primary individuals who interacted with the ITCs. Additionally, the number of focus groups was limited at both time points, but the consistency of results suggested that saturation had been reached regarding the topics explored.

## Conclusion

Results from our study highlight the importance of gathering process (as opposed to only outcome) data throughout the course of a study. The first we became aware that the curtains were not performing as expected was during focus group discussions conducted within 6 months of installing ITCs in people’s homes. The qualitative work that accompanied the KAP surveys allowed us to detect a problem in the performance of the intervention and address it through re-treatment of the ITCs. It also helped us further interpret survey data regarding whether people would recommend the ITCs in the future.

Initial failure of the ITCs demonstrates that their long-term effectiveness needs to be enhanced, particularly to address the reported disadvantages about re-treatment and allergic reactions. We strongly feel that people’s use and recommendations would have been more favorable had we not had effectiveness problems within a few months of ITC installation. Ideally, ITCs should not require re-treatment or cause allergic reactions. When re-treatment is necessary, the process should be conducted in a way that is minimally intrusive to the participants. In our study it was important for us to keep track of each ITC and if it was being used correctly. Flexibility, however, in allowing participants to move their ITCs to other locations should have been considered. Once the ITCs were placed in homes, and participants started interacting with them, they determined that some locations were not as convenient as others. Similarly, alternative methods for hanging the ITCs should be considered (e.g., hanging ITCs in locations that are less likely to result in accidental physical contact, like along a wall) to reduce adverse effects experienced by participants.

Participants in our study reported that they would be willing to continue hanging the ITCs in the home even if they were no longer effective. Feeling like the curtains look good in the home, or have alternative uses such as added privacy, can be helpful in ensuring that the curtains are consistently hung properly in order to provide protection against mosquitoes.

From an acceptability perspective, ITCs have great potential as a dengue vector control tool. They are well-liked by users because of their effectiveness and for aesthetic reasons, and because they require little behavioral input from the users. When functioning optimally, their impact on dengue vectors and DENV transmission will be important to quantify.

## Abbreviations

*Ae. aegypti*, *Aedes aegypti;* FGD, focus group discussion; ITC, insecticide-treated curtain; ITM, insecticide-treated material; KAP, knowledge, attitudes, practices; NAMRU-6, U.S. Naval Medical Research Unit-Six
